# Genetics and Extracellular Vesicles of Pediatrics Sleep Disordered Breathing and Epilepsy

**DOI:** 10.3390/ijms20215483

**Published:** 2019-11-04

**Authors:** Abdelnaby Khalyfa, David Sanz-Rubio

**Affiliations:** 1 Department of Pediatrics, Section of Sleep Medicine, The University of Chicago, Chicago, IL 60637, USA; 2 Department of Child Health and the Child Health Research Institute, University of Missouri School of Medicine, Columbia, MO 65201, USA; dsanzrubio@health.missouri.edu

**Keywords:** sleep, sleep disorders, epilepsy, pediatric, *CYP2C19*, CYP45, drug metabolism, phenobarbital, GABA, GABARA, GABARB, GABARC, metabolic function, extracellular vesicles, exosomes

## Abstract

Sleep remains one of the least understood phenomena in biology, and sleep disturbances are one of the most common behavioral problems in childhood. The etiology of sleep disorders is complex and involves both genetic and environmental factors. Epilepsy is the most popular childhood neurological condition and is characterized by an enduring predisposition to generate epileptic seizures, and the neurobiological, cognitive, psychological, and social consequences of this condition. Sleep and epilepsy are interrelated, and the importance of sleep in epilepsy is less known. The state of sleep also influences whether a seizure will occur at a given time, and this differs considerably for various epilepsy syndromes. The development of epilepsy has been associated with single or multiple gene variants. The genetics of epilepsy is complex and disorders exhibit significant genetic heterogeneity and variability in the expressivity of seizures. Phenobarbital (PhB) is the most widely used antiepileptic drug. With its principal mechanism of action to prolong the opening time of the γ-aminobutyric acid (GABA)-A receptor-associated chloride channel, it enhances chloride anion influx into neurons, with subsequent hyperpolarization, thereby reducing excitability. Enzymes that metabolize pharmaceuticals including PhB are well known for having genetic polymorphisms that contribute to adverse drug–drug interactions. PhB metabolism is highly dependent upon the cytochrome P450 (CYP450) and genetic polymorphisms can lead to variability in active drug levels. The highly polymorphic *CYP2C19* isozymes are responsible for metabolizing a large portion of routinely prescribed drugs and variants contribute significantly to adverse drug reactions and therapeutic failures. A limited number of *CYP2C19* single nucleotide polymorphisms (SNPs) are involved in drug metabolism. Extracellular vesicles (EVs) are circular membrane fragments released from the endosomal compartment as exosomes are shed from the surfaces of the membranes of most cell types. Increasing evidence indicated that EVs play a pivotal role in cell-to-cell communication. Theses EVs may play an important role between sleep, epilepsy, and treatments. The discovery of exosomes provides potential strategies for the diagnosis and treatment of many diseases including neurocognitive deficit. The aim of this study is to better understand and provide further knowledge about the metabolism and interactions between phenobarbital and *CYP2C19* polymorphisms in children with epilepsy, interplay between sleep, and EVs. Understanding this interplay between epilepsy and sleep is helpful in the optimal treatment of all patients with epileptic seizures. The use of genetics and extracellular vesicles as precision medicine for the diagnosis and treatment of children with sleep disorder will improve the prognosis and the quality of life in patients with epilepsy.

## 1. Sleep Disordered Breathing (SDB)

Pediatrics SDB describes a wide spectrum of respiratory disorders which are characterized by partial or complete obstruction of the upper airways ranging from primary snoring, the mildest clinical manifestation, to obstructive sleep apnea syndrome (OSAS): complete obstruction of the upper airways with cessation of airflow during sleep [1]. Pediatrics sleep disordered breathing are very important during childhood because they are associated with developmental and cognitive deficits and with reduced learning or social skills [2]. The prevalence of OSAS is estimated between 2–5%, and the choice of treatment is made on an individual basis which depends on the following factors: child’s age, polysomnographic pattern, comorbidities, and complications related to OSAS [3,4]. Surgical treatment, including tonsillectomy and adenoidectomy, is the first choice of treatment in children with adenotonsillar hypertrophy and OSAS. However, continuous positive airway pressure (CPAP) application remains a viable option for children not eligible for surgery, or in cases of residual disease [5,6]. Recently, the effect of CPAP in children with OSAS was reported the results suggested that children with OSAS who were treated with CPAP therapy experienced significant improvement in their pedestrian safety, as measured in a virtual pedestrian environment, following adherence to CPAP therapy [6].

Most children with OSAS snore and have difficulty breathing during sleep. Recently childhood sleep and its relation to sleep quantity, quality, and variability in a clinic sample of mothers of toddlers and preschool-age children with epilepsy were reported [7]. Sleep disturbances are more frequently reported in children with epilepsy [8,9], and this can significantly affect the quality of life and functionality of children in general and those with comorbid neurological diseases in particular [10]. Children with epilepsy syndromes are known for their nocturnal activation such as benign Rolandic epilepsy and benign occipital epilepsy of childhood. In addition, sleep disorders can adversely affect seizure control and sleep deprivation leads to activation of seizures even in healthy individuals [11].

## 2. Pediatrics Sleep Disorders and Epilepsy

Pediatrics sleep-disordered breathing (P-SDB) is one of the most common behavioral problems in childhood, and sleep disorders are an important trigger for epileptic seizures [12]. Sleep-disorders have an even greater prevalence in children with epilepsy and are one of the most common comorbid conditions in childhood epilepsy [13]. Furthermore, the incidence of sleep apnea in pediatric epilepsy is type-dependent as is shown a study in which the prevalence of sleep apnea in children with refractory epilepsy was higher compared with children with mild epilepsy [14]. In addition, a correlation between pediatric epilepsy severity and aberrant sleep has been described, and the correct management of a child with epilepsy and sleep disorder is very crucial [15]. For example, performing an accurate diagnosis may be not simple and different tools are needed. First, it is important to recognize the contribution of the parent with different questionnaires that have been shown as good predictors of sleep disorders such as the Children’s Sleep Habits Questionnaire, the Pediatric Sleep Questionnaire or the Sleep Disturbance Scale for Children [13,16,17,18]. Second, the clinical examination of the patient could suggest the presence of sleep apnea due to tonsillar hypertrophy, although it is not a definitive diagnosis [17]. Finally, to confirm the sleep disorder, an overnight polysomnography (PSG) is required and necessary [18,19]. This test will also allow determination of SBD such as sleep apnea, hypoventilation, central sleep apnea, insomnia or periodic limb movement disorder [18].

## 3. Epilepsy

Epilepsy is defined by the International League Against Epilepsy (ILAE) as a chronic neurological disease (the most common neurologic disease in children) characterized by recurrent seizures [20]. Epilepsy subjects undergo repeated seizures that involve physical and/or behavioral changes that interrupt their normal activities [21]. About 66% of children with epilepsy achieve seizure control using a single antiepileptic drug (a.k.a., monotherapy) [22]. In general, epilepsy can be classified based upon: (a) clinical features of the seizure episodes; (b) etiology; (c) anatomic site of seizure origin; (d) tendency to spread to other structures in the brain; and, (e) temporal patterns. Several factors should be measured to characterize the type and severity of epilepsy, including seizure frequency and duration. The impact of seizures, treatments (e.g., medications, surgery) and treatment side effects on children’s daily life activities should also be considered to get a more complete picture of the patient’s condition. For example, O’Donohoe (1983) suggested that restrictions imposed on a child with epilepsy are typically in proportion to the severity of their epilepsy, and children with intractable or refractory epilepsy, whose seizures are not well controlled by treatment, are typically considered to have a more severe form of epilepsy [23]. For some children with epilepsy, repetitive seizures may negatively impact their quality of life, education employment and they may be at elevated risk of early mortality [24]. Epilepsy is a common neurological disorder, that affects 1–3% of the general population and ~0.6% of the pediatric population [25]. The genetics of epilepsy is complex and typically involves the interaction between several or many genes. Furthermore, genetic epilepsy disorders exhibit significant genetic heterogeneity, variable penetrance and expressivity of seizures [26], which in some cases, may reflect mosaic gene expression.

The total cost per year for medical expenditure and informal care for patients with epilepsy in USA is estimated to be $15.5 billion in direct costs (medical) and indirect costs (lost or reduced earnings and productivity) **(**https://www.takeonepilepsy.com/). The underlaying of pediatrics epilepsy is unknown. In children, the most common causes of secondary (or symptomatic) epilepsy are: (a) brain damage due to pre-natal or prenatal injuries (including hypoxic events and/or trauma during birth); (b) congenital brain defects or genetic conditions that are associated with brain malformations; (c) traumatic brain injury, (d) stroke resulting in hypoxic injury to the brain; (e) infections in the central nervous system such as meningitis, encephalitis, neurocysticercosis; (f) some genetic syndromes; and, (g) brain tumor(s) (e.g., tuberous sclerosis) (http://www.who.int/news-room/fact-sheets/detail/epilepsy).

Individual genetic variability, along with the increased presence of comorbidities (which if pharmacologically treated, may increase the potential for interactions with other medications) may contribute to unpredictable fluctuations in drug serum concentrations, particularly in pediatric populations. For example, children with epilepsy may have comorbid conditions that require specific pharmacological therapies that can interfere with anti-epileptic drug treatment. Drug interactions can occur at a pharmacokinetic level—involving the absorption, distribution, and elimination of the drug—as well as at a pharmacodynamics level, resulting in synergistic or antagonist effects at the site of action [27]. More importantly, pharmacokinetic interactions involve the induction and/or inhibition of drug metabolism. For example, carbamazepine, phenytoin, and phenobarbital are potent inducers of liver enzymes and can reduce the efficacy of co-administered drugs, whereas other antiepileptic drugs can inhibit liver enzymes, reducing the clearance of co-administered drugs and thereby increasing their efficacy and the likelihood of toxic effects [28].

### 3.1. Epilepsy Treatments

Epilepsy treatment typically requires lifelong adherence to anti-epileptic drug (AED) regimes, yet AED treatment fails to provide seizure control in about ~33% of patients. For many individuals with medically intractable seizures, surgery is an option and while typically successful, about ~33% of individuals with epilepsy continue to experience seizures after surgery [29,30]. There are several AEDs that have been used to treat epilepsy patients including: Acetazolamide, Brivaracetam, Carbamazepine, Clobazam, Clonazepam, Eslicarbazepine acetate, Ethosuximide, Gabapentin, Lacosamide, Lamotrigine, Levetiracetam, Oxcarbazepine, Perampanel, Phenobarbital, Phenytoin, Piracetam, Pregabalin, Primidone, Rufinamide, Stiripentol, Tiagabine, Topiramate, Valproic acid, Vigabatrin, Zonisamide (httpns://www.epilepsysociety.org.uk/list-anti-epileptic-drugs#.XASMj2hKg2w). Although there are numerous alternative treatment choices for epilepsy, including vagus nerve stimulation, surgery the ketogenic diet, AEDs remain the first choice for seizure control as 66% of epilepsy patients experience seizure-freedom after taking an AED [31]. However, AEDs should be used and dosed carefully because of the potential for drug–drug interactions and side effects, such as dizziness, drowsiness, mental slowing, skin rashes, liver toxicity, movement and behavioral disorders, as well as metabolic disturbances, such as weight loss/gain, metabolic acidosis, and nephrolithiasis [32].

In children, current evidence indicates that adherence to AED treatment varies among children with epilepsy between 22.1% and 96.5% [33]. AED noncompliance also leads to an increase burden on inpatient and emergency department services. Moreover, it also affects the family members socially, economically and psychologically [34,35]. In this review, we focus on the AED phenobarbital as it has a long history of successfully treating epilepsy since the early decades of the 20th century. The phenobarbital advantages are its long history of use, low cost and effectiveness; these attributes may especially provide several advantages toward seizure control in populations in developing nations.

### 3.2. Pediatrics Sleep Disorders and Epilepsy Treatment

The treatment of both pathologies (P-SDB and epilepsy) can affect each other. On the one hand, the treatment of sleep apnea leads to reduce seizures [36]. Melatonin is a hormone-related with the regulation of sleep-wake cycles, and there is some controversy about using this drug in children with epilepsy due to its possible adversary effect [17,37]. However, another study has shown the improvement of both SBD and epileptic seizures after melatonin uptake [38]. On the other hand, the effects of AEDs in SBD must be analyzed with caution, and some studies have described that the polytherapy gets worse the SBD [14]. Several studies have shown a direct effect of specific AEDs in sleep disorder and day sleepiness was increased in patients taking carbamazepine, phenytoin, valproic acid, and phenobarbital [39]. In addition, phenobarbital and phenytoin have been reported to decrease rapid eye movement (REM) sleep [39,40], while benzodiazepines are known to decrease sleep latency and the amount of both slow-wave and REM sleep [39]. These benzodiazepines could have relaxant effects on muscles related to sleep breathing while others as pregabalin and valproate could worsen sleep apnea due to the weight gain. Other AEDs such as gabapentin [39] and pregabalin have been described to increase slow-wave sleep and reduce arousals [41].

### 3.3. Phenobarbital

Phenobarbital (5-ethyl-5-phenyl-1,3-diazinane-2,4,6-trione) is an AED recommended by the World Health Organization (WHO) to treat certain types of epilepsy in developing nations [42]. PhB was discovered to have anti-seizure properties by Alfred Hauptmann, who originally administered it to epilepsy patients as a hypnotic whereby he discovered it could interrupt their seizures [43]. Over the last 20 years, there have been a lot of new AEDs introduced and today, at least 20 drugs are licensed for current use for epilepsy. Phenobarbital (PhB) is the most widely used AED and remains a popular medication in many developing nations. Despite the development of new AEDs, PhB holds a unique position in the therapeutic armamentarium as it is still the most widely prescribed treatment for epilepsy worldwide [44]. For example, Dutch studies showed that phenobarbital is still the fourth most commonly prescribed antiepileptic for pediatric epilepsy, with relatively stable prescription rates over the past 10 years [45]. In Germany, PhB is also the most commonly used conventional AED among children aged < 2 years, accounting for 40.1% of AED use, falling to 2.6% (fourth place) in the 12–17 year age group [46]. In Africa, Phenobarbital is widely used as a low cost and effective addition to the antiseizure arsenal, particularly in pediatric epilepsies [47]. Side effects are much more likely to be troublesome when high doses are given and when a high dose is given at the onset of treatment to get seizures under control. To avoid side effects, it may be necessary to increase the dosage very slowly over several months (https://www.epilepsy.com/medications/phenobarbital). Further studies are necessary to evaluate the dose-dependent sleep effects of antiepileptic drugs and nondrug treatments independent of the improvement of epilepsy, and to identify if these changes are clinically significant [48].

The mechanism by which PhB is thought to suppress seizures, is by prolonging the open time of *γ*-aminobutyric acid (GABA)-A receptor ion channels thereby increasing the duration of chloride anions influx into neurons, causing subsequent hyperpolarization, reducing the excitability and suppression of seizure activity [49].

Thus, PhB is thought to activate the GABA_A_ receptor directly and the actions of PhB through these effects can promote sleep, reduce anxiety, have anesthetic effects and suppress or prevent partial tonic–clonic and generalized seizures [50].

**Figure 1:**
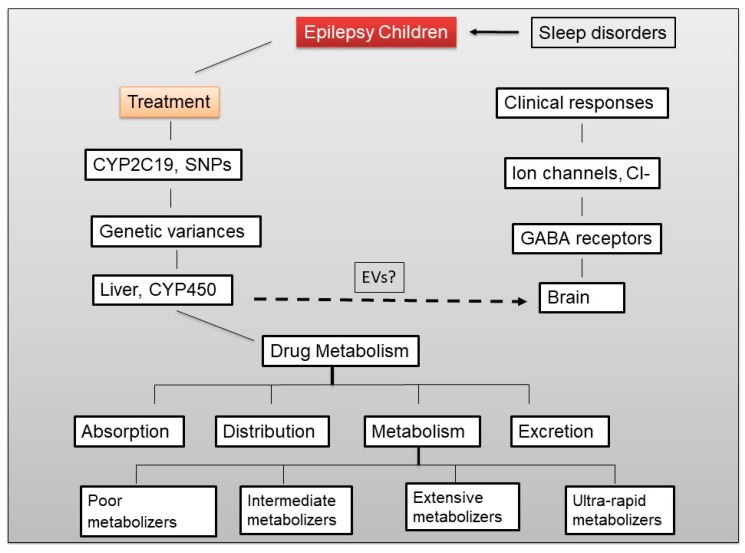
Diagram illustrates the effects of sleep disorders and epilepsy treatment on γ-aminobutyric acid (GABA) receptors (A, B & C), *CYP2C19* gene and drug metabolism.

Approximately 25% of a PhB dose is not metabolized and excreted unchanged in the urine [51]. The majority of PhB that gets absorbed metabolized in the liver into two inactive metabolites, *p*-hydroxyphenobarbital via aromatic hydroxylation, and 9-D-glucopyranosylphenobarbital via glucosidation. However, there is large inter-individual variability in the proportions of these metabolites that individuals generate [52]. PhB is water-soluble, metabolized by the liver, while its metabolites are primarily expelled through the kidneys [43]. PhB is known to induce the expression of cytochrome p450 enzymes in the liver, and the induction of these enzymes are likely to speed up the metabolism of not only PhB, but also estrogens and progestins (Figure 1).

## 4. Gamma-Aminobutyric Acid (GABA) Receptors

GABA is a major inhibitory neurotransmitter involved in regulating inhibitory synaptic communication between brain cells. The role of GABA is to inhibit or reduce the activity of neurons and networks in the brain. GABA acts via two classes of receptors: GABA_A_ and GABA_B_ receptors. GABA_A_ receptors are ionotropic while GABA_B_ receptors are metabotropic. The binding of GABA to postsynaptic GABA receptors opens an ion channel that is chloride-selective and typically carries negatively charged chloride ions into neurons, making them less-excitable [53]. GABA_A_ receptors have five subunits that originate from seven families containing multiple subunits but most commonly assemble with two alpha subunits, two beta subunits, and one gamma [54,55,56]. When GABA binds to a GABA_A_ receptor, ligand gated ion channel opens, allowing chloride, a negatively charged ion, into the neuron hyperpolarizing the cell. This hyperpolarization makes it more difficult for the neuron to reach action potential threshold.

## 5. Identifying GABA_A_ Receptor Gene Networks

**Figure 2:**
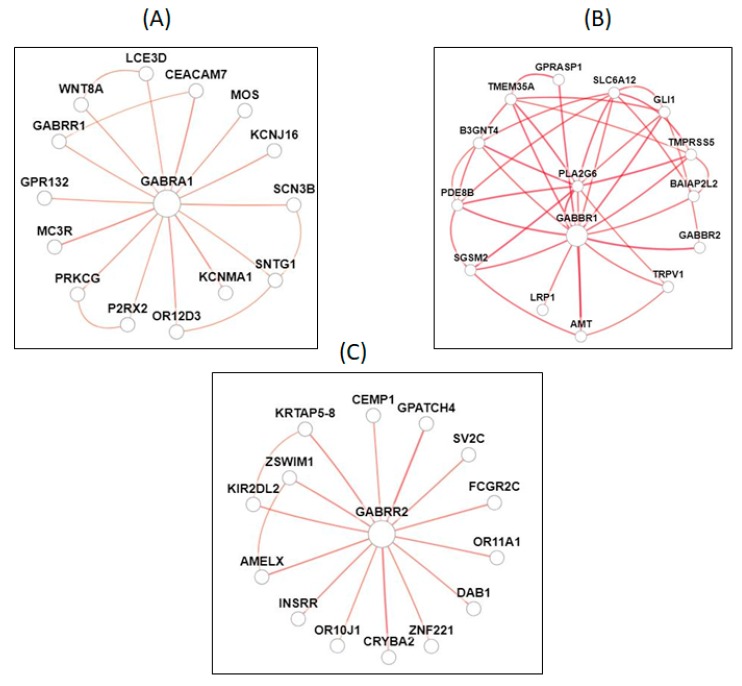
Potential networks of GABA receptors and association with ion channels. Panel (**A**) is the GABA receptor A 1 (*GABAR1*) and gene networks of chloride transport genes. Panel (**B**) is GABA receptor B (*GABRB1*) and networks of potassium transport. Panel (**C**) is GABA receptor C (*GABA-A*) and networks of chloride transport.

GABA_A_ receptors are ligand gated, chloride selective ion channels formed from various combinations of proteins and subunit gene families. In cases where there is a dysfunction of these subunits, the ion channel gating expression and trafficking of the GABA_A_ receptor to the cell surface can be affected [57]. The gene networks of *GABAR1* and chloride transporter are shown in Figure 2A, and the genes that are involved in these networks include the following: potassium calcium-activated channel subfamily M alpha (*KCNMA1*), melanocortin 3 receptor (*MC3R*), carcinoembryonic antigen related cell adhesion molecule (*CEACAM7*), olfactory receptor family 12 subfamily D member 3 (*OR12D3*), sodium voltage-gated channel beta subunit 3 (*SCN3B*), protein kinase C gamma (*PRKCG*), potassium voltage-gated channel subfamily J member 16 (*KCNJ16*), MOS proto-oncogene, serine/threonine kinase (*MOS*), Wnt family member 8A (*WNT8A*), late cornified envelope 3D (*LCE3D*), gamma-aminobutyric acid type A receptor rho1 subunit (*GABRR1*), G protein-coupled receptor 132 (*GPR132*), purinergic receptor P2X 2 (*P2RX2*), syntrophin gamma 1 (*SNTG1*), and basal body orientation factor 1 (*BBOF1*).

Genes encoding voltage-gated Na^+^, K^+^, Ca^2+^, and Cl^−^, ion channels are considered to be the most important class of genes that are associated with epilepsy [58,59]. It has been reported that GABA type-A receptor (*GABRA1*) is a major genetic cause of heritable epilepsies in humans [60]. These receptors are localized to postsynaptic sites in the brain. Synaptic transmission leads here to the release of GABA, that can open GABA_A_ receptor chloride channels, thereby increasing chloride conductance, leading to brief hyperpolarization of a depolarized membrane [61]. In addition to the central nervous system, GABA_A_ receptors have also been found in the liver, smooth airway muscles of the lung, and in several immune cell types [62,63,64].

We will highlight very briefly recent literature regarding each of these receptors in terms of their sequences homology using Kyoto Encyclopedia of Genes and Genomes (KEGG) pathways and Gene Ontology (GO), please see Table 1. GABA_A_ receptors consist of proteins with several subunit classes: alpha, beta, gamma, delta and rho. The GABA_A_ gene is located on chromosome 5: 161,847,063–161,899,981 with RefSeq (NM_000806.5), and protein ID (NP_000797.2), has 20 splices variants. GABA_A_ receptors are modulated by post-translational modification, e.g., several different protein kinases phosphorylate specific amino acid resides on specific receptor subunits and thereby modulate channel activity [65].

Next, we checked the sequence homology of GABAR1 (NM_000806.5), GABBR1 (NM_000812), and GABBR2, GABA-C, (NM_002043.4) against human genome sequences, KEGG pathways, and Gene Ontology as shown in Table 1.

The genes network of GABABR1 and potassium ion transport are showed in Figure 2B. They are linked to the following genes: gamma-aminobutyric acid type B receptor subunit 2 (*GABBR2*), aminomethyltransferase (*AMT*), phospholipase A2 group VI (*PLA2G6*), phosphodiesterase 8B (*PDE8B*), GLI family zinc finger 1 (*GLI1*), G protein-coupled receptor associated sorting protein 1 (GPRASP1), transmembrane protein 35A (*TMEM35A*), small G protein signaling modulator 2 (*SGSM2*), transmembrane serine protease 5 (*TMPRSS5*), solute carrier family 6 member 12 (SLC6A12), BAI1 associated protein 2 like 2 (*BAIAP2L2*), UDP-Glc NAc:betaGal beta-1,3-*N*-acetylglucosaminyltransferase 4 (*B3GNT4*), transient receptor potential cation channel subfamily V member 1 (*TRPV1*), LDL receptor related protein 1 (*LRP1*), and mitogen-activated protein kinase 10 (*MAPK10*).

GABAA-rho receptors, previously known as GABA_C_ receptors, (named as GAPA-A) are Cys-loop ionotropic ligand-gated ion channels mediating the fast-synaptic inhibitory effects of GABA. GABA-A receptors are the newly identified member of the GABA receptor family. They are also linked to chloride channels, with distinct physiological and pharmacological properties. This receptor contains 465 amino acids, NM_002043.4, NP_002034.3, chromosome 6: 89,257,208–89,315,299. The gene networks of GABA_C_ or GABBR2 with chloride ion transports are illustrated in Figure 2C. This GABA_C_ (GABA-A) is associated in the following gene networks: G-patch domain containing (GPATCH4), crystallin beta A2 (*CRYBA2*), amelogenin X-linked (*AMELX*), keratin associated protein (*KRTAP5*-8), olfactory receptor family 11 subfamily A member 1 (*OR11A1*), killer cell immunoglobulin like receptor, two Ig domains and long cytoplasmic tail (*KIR2DL2*), Fc fragment of IgG receptor IIc (*FCGR2C*), zinc finger SWIM-type containing (*ZSWIM1*), synaptic vesicle glycoprotein (*SV2C*), insulin receptor related receptor (*INSRR*), reelin adaptor protein (*DAB1*), zinc finger protein (*ZNF221*), cementum protein (*CEMP1*), olfactory receptor family 10 subfamily J member 1 (*OR10J1*), and regulatory factor X4 (*RFX4*).

## 6. Cytochromes P450 (CYP450) and *CYP2C19* Gene

Human CYP450 are primarily membrane-associated proteins located either in the inner membrane of mitochondria or in the endoplasmic reticulum of cells [66]. CYP450 enzymes have potential uses in the synthesis and discovery of drugs, as well as drug development. CYP450 enzymes metabolize thousands of endogenous and exogenous chemicals. CYP450 constitutes the major enzyme family capable of catalyzing the oxidative biotransformation of most drugs and other lipophilic xenobiotics and are therefore of particular relevance for clinical pharmacology [67], primarily found in liver cells but they are also located in cells throughout the body. Some CYPs metabolize only one (or very few) substrates, such as CYP19 (aromatase), while others may metabolize multiple substrates. These CYP enzymes are present in most tissues of the body, and play important roles in hormone synthesis and breakdown of each of estrogen and testosterone synthesis and metabolism, cholesterol synthesis, and vitamin D metabolism [68].

CYP450 enzymes are bound to membranes within a cell and contain a heme pigment that absorbs light at a wavelength of 450 nm when exposed to carbon monoxide. There are more than 50 CYP450 enzymes, but the CYP1A2, CYP2C9, CYP2C19, CYP2D6, CYP3A4, and CYP3A5 enzymes metabolize 90 percent of drugs [69]. CYP450 enzymes play a role in the synthesis of many molecules including steroid hormones, certain fats (cholesterol and other fatty acids), and acids used to digest fats (bile acids). Up-to-date, there are approximately 60 CYP450 genes in humans (https://ghr.nlm.nih.gov/primer/genefamily/cytochromep450).

Hepatic CYP450 mutations can alter systemic drug metabolism and distribution, however, the possibility that mutated or polymorphic variants of CYP450 exist in the brain is not well studied (Figure 1). Early studies in epilepsy have explicitly demonstrated that patients who respond to early therapy have a better prognosis, while patients who fail to respond to the first- or second-line antiepileptic drugs are most likely to develop intractable epilepsy [70,71,72]. In the report we will only discuss the *CYP2C19* gene as outlined in Figure 1.

The CYP450, family 2, subfamily C, polypeptide 19 (*CYP2C19* gene) is located within a cluster of CYP450 genes (*CYP2C19*) on chromosome 10q23.33 with reference sequence number (NM_000769) and ensemble accession number ENST00000371321.8. *CYP2C19* is expressed in the liver and to a lesser extent in the small intestine [73], contributing to the metabolism of a large number of clinically relevant drugs and drug classes including phenytoin and phenobarbital [74,75]. The *CYP2C19* gene is part of the CYP2C cluster comprising four genes (*CYP2C8, CYP2C9, CYP2C18* and *CYP2C19*). This *CYP2C19* gene encompasses 90,637 bases and is composed of nine exons encoding 490 amino acids. The position of exons and introns of this gene is shown in Table 2.

We checked nucleotide human sequence homology of *CYP2C19* (NP_000760.1) with National Institute of Health (NIH) databases (https://www.ncbi.nlm.nih.gov/), and we found that 84% of *CYPC29* (NP_000760.1), 81% of *CYP2C18* isoform 2 precursor (NP_000762.2), 81% of *CYP450* 2C8 isoform c (NP_000763.1), 79% of *CYP450* 2C8 isoform b (NP_001185783.1), 78% *CYP450* 2C8 isoform a precursor (NP_000761.3), 58% *CYP450* 2E1 precursor (NP_000764.1), 56% *CYP450* 2F1 isoform X7 (XP_016881875.1), and 48% *CYPP450* 2F1 isoform X4 (XP_011524855.1).

**Figure 3:**
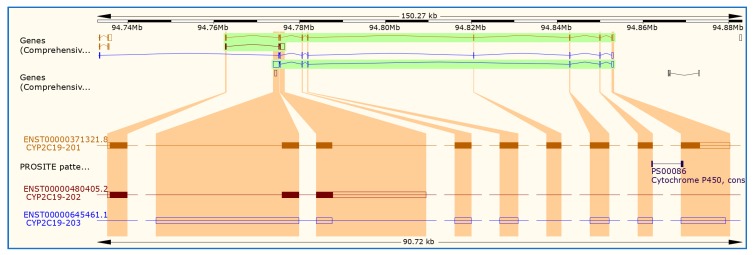
Promoter regions of *CYP2C19* transcripts in the human genome. As shown, there are three transcripts for *CYP2C19* including CYP2C19-201 (ENST00000371321.3) composed of 1901 nucleotides and protein coding 490 amino acids (aa), CYP2C19-002 (ENST00000464755.1), composed of 2395 nucleotides and no protein coding, and CYP2C19-003 (ENST00000480405.1) composed of 1417 and no protein coding.

Understanding the biological function of *CYP2C19* gene can provide a good foundation for subsequently interpreting the potential influence of genetic associations on a disease phenotype and so can greatly support any identified genetic association. Functional studies can include those investigating changes in gene expression, splicing and protein function and should be performed in conjunction with genetic studies to improve data interpretation and strengthen data analysis. Another essential endpoint is clinical phenotype in this case pharmacological phenotypes. For example, promoter region variants can influence the enzyme activity by altering the gene expression [76]. A promoter is a region of DNA where transcription of a gene initiates. Promoters are adjacent and typically upstream (5′) of the sense strand of the regulated gene. Promoters are a vital component of expression vectors because they control the attachment of RNA polymerase to DNA and are directly responsible for the amount of transcript generated. Potential binding sites for the transcription factors such as GATA proteins, progesterone receptor, TATA box elements and nuclear factors in *CYP2C19* promoter region has been described [77]. The promoter region controls gene expression by the interaction of cis-acting elements spanning through the promoter region with the transcription factors. This *CYP2C19* gene (RefSeq accession number NM_000769.4) has three promoter regions (Figure 3). As shown in Figure 3, *CYP2C19*-201 has nine exons and eight introns (Table 1) and consists of 490 amino acids, while CYP2C10-202 has 162 amino acids, however, *CYP2C19*-203 does not produce protein products. The sequences for all promoters in *CYP2C19* gene are as follows: *CYP2C19*-201 identified in chromosome position at 94762681 (ttaattagcatggagtgttataaaaagcttggagtgcaagctcacggttGTCTTAACAAG), *CYP2C19*-202 chromosome position 94842926 (ccggagcccctgcatgcaggacaggggccacatgccctacacagatgctGTGGTGCACGA), and *CYP2C19*-203 chromosome position 94775489 (ctcatgacgctgcggaattttgggatggggaagaggagcattgaggaccGTGTTCAAGAG)

In addition, we identified the following KEGG pathways of *CYP2C19* which include arachidonic acid metabolism, organism-specific biosystem, chemical carcinogenesis, organism-specific biosystem, drug metabolism (cytochrome P450), organism-specific biosystem, linoleic acid metabolism, organism-specific biosystem, metabolic pathways, organism-specific biosystem, and serotonergic synapse. Next, we used the advantage of the gene ontology (GO) project that provides the most comprehensive resource currently available for computable knowledge regarding the functions of genes and gene products. For the cellular component, the *CYP2C19* gene is found in different part of the cells: cytoplasm (GO:0005737), endoplasmic reticulum (GO:0005783), endoplasmic reticulum membrane (GO:0005789), membrane (GO:0016020), and organelle membrane (GO:0031090). The biological Process are organic acid metabolic process (GO:0006082), xenobiotic metabolic process (GO:0006805), steroid metabolic process (GO:0008202), and monoterpenoid metabolic process (GO:0016098), and drug metabolic process (GO:0017144). The molecular function includes monooxygenase activity (GO:0004497), iron ion binding (GO:0005506), arachidonic acid epoxygenase activity (GO:0008392), steroid hydroxylase activity (GO:0008395), and oxidoreductase activity (GO:0016491).

**Figure 4:**
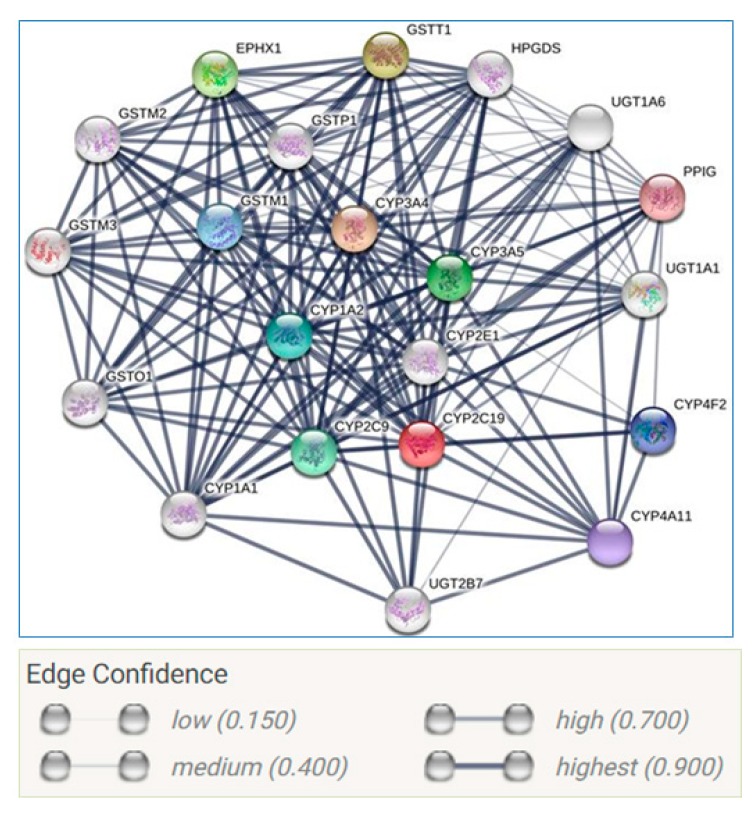
Gene network for the *CYP2C19* gene and selected interacting protein using STRING software utilizing version 10.5. STRING is a biological database and web resource of known and predicted protein–protein interactions, and imports data from experimentally derived protein–protein interactions through literature curation. Furthermore, STRING also stores computationally predicted interactions using text mining of scientific texts, interactions computed from genomic features, and interactions transferred from model organisms based on orthology. All predicted or imported interactions are benchmarked against a common reference of functional partnership as annotated by KEGG (Kyoto Encyclopedia of Genes and Genomes).

Next, we used STRING software to build a gene network of the *CYP2C19* gene and other neighbor genes using https://string-db.org/ as shown in Figure 4.

**Figure 5:**
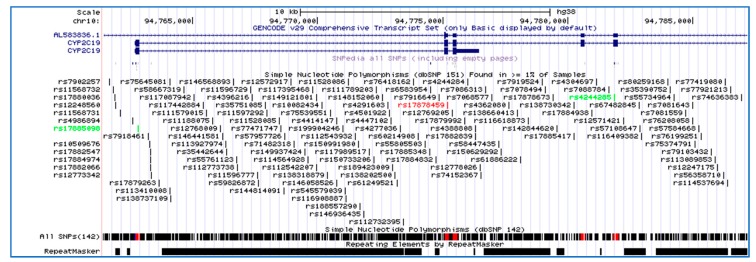
Single nucleotide polymorphisms (SNPs) of the *CYP2C19* gene was identified using the University of California, Santa Cruz (UCSC) genome Browser on Human (GRCh37/hg19) assembly from position 94,765,00 to 94,785,00 on chromosome 10.

This network shows high interaction between *CYP2C19* and other genes such as glutathione S-transferase mu 1 (*GSTM1*), glutathione S-transferase mu 2 (muscle), glutathione S-transferase mu 3 (brain), glutathione S-transferase theta 1 (*GSTT1*). The advantage of complete human genome project can lead to the identification of single nucleotide polymorphisms (SNPs) as the most common form of genetic variation [78,79]. SNPs comprise a large set of bi-allelic genomic variants that are found approximately every 100–300 bp throughout the genome, although less frequently in protein coding DNA sequences. Genetic polymorphisms of CYP450 enzymes mainly influence drug metabolism, disposition and elimination and lead to alteration of pharmacokinetic parameters resulting in changes in drug response as well as adverse drug reactions [80]. Next, we used genome at USSC to find out the *CYP2C19* tagged SNPs (https://genome.ucsc.edu/cgi-bin/hgGateway). The tagged SNPs associated with the *CYP2C19* gene are shown in Figure 5. A tagged SNP is a representative SNP in a region of the genome with high linkage disequilibrium that represents a group of SNPs called a haplotype.

**Figure 6:**
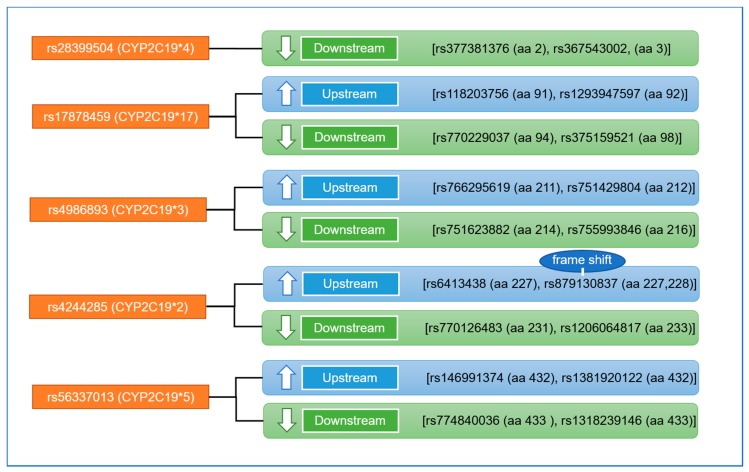
Five single nucleotide polymorphisms (SNPs) of the *CYP2C19* gene that are involved in drug metabolism. We identified two upstream and two downstream for each of those SNPs and their location in the gene sequence.

In this report, we will focus only on *CYP2C19* SNPs that has been reported to be associated with drug metabolism as shown in Table 2. We identified the position of each amino acid for each SNPs and the upstream SNPs and downstream SNPs including SNPs (rs28399504 (CYP2C19*4) located on amino acid number 1 (valine), rs17878459 (CYP2C19*17), located on amino acid 92, (aspartic, D), rs4986893 CYP2C19*3 located on amino acid number 212 (NA), rs4244285 (CYP2C19*2) located on amino acid 227 (proline), and rs56337013 (CYP2C19*5) located on amino acid number 433 (tryptophan, W). Figure 6 shows the position of the two up-streams and down-streams SNPs.

In general terms, people carrying two loss-of-function *CYP2C19* alleles (e.g., CYP2C19*2, CYP2C19*3 or CYP2C19*4) are considered to be poor metabolizers (PMs) with deficient enzyme activity. Individuals with single loss-of-function allele and a wild-type allele (CYP2C19*1) are intermediate metabolizers (IMs), whereas two wild-type are extensive metabolizers (EM). People that are homozygous or heterozygous for CYP2C19*17 alleles are typically considered to be ultrarapid metabolizers (UM)*,* supposing loss-of-function alleles are not present. It has been suggested that inherited genetic variation in *CYP2C19* and its variable hepatic expression can contribute to inter-individual phenotypic variability in *CYP2C19*-substrate metabolism [75]. *CYP2C19* proteins can catalyze many reactions involved in drug metabolism and synthesis of cholesterol, steroids and other lipids, and this protein localizes to the endoplasmic reticulum and is known to metabolize many drugs. It has been suggested that polymorphism within this gene is associated with variable ability to metabolize mephenytoin, known as the poor metabolizer and extensive metabolizer phenotypes [81,82]. The genotype has been suggested *CYP2C19* to affect the metabolism of several [83]. It has been reported that CYP2C19*2 and *3 mutant alleles caused decreased hydroxylation of phenytoin in vivo, whereas the mutant alleles of *CYP2C19* played only a minor role in the metabolism of phenytoin in subjects [84]. In additions, others have also reported strong association between *CYP2C9* allelic variants and phenytoin dose requirement [85]. Interestingly, the loss-of-function polymorphisms in CYP genes often affect splicing and expression, rather than transcription or protein structure [86]. The genetic polymorphisms of *CYP2C19* have been associated with variable responses of drugs with clinical relevance, by affecting drug metabolic clearance that subsequently contribute to variabilities in pharmacodynamics (PD) that include both efficacy and safety [83].

## 7. Mechanism of Drug Metabolism

Drug metabolism is defined as the biochemical modification of one chemical form to another, occurring usually through enzymatic systems. One of the major contributors to the inter-individual variability in drug response is the difference in the capacity to metabolize the drugs, which translates from genetic SNPs or by drug interactions involving inhibition and induction of drug metabolizing enzymes. Moreover, drug metabolisms can be classified into two categories: phase I (oxidation, reduction, and hydrolysis) and phase II (conjugation). Oxidative processes are mediated primarily by the CYP450 family of enzymes, whereas conjugation reactions are conducted largely by the enzyme uridine 5′-diphospho-glucuronyltransferase (UGT) [87]. The activity of CYP and UGT can be influenced by genetic, environmental (exogenous), and endogenous factors resulting in significant variation among individuals in drug metabolism [88].

The CYP450 enzymes activity have four recognizable metabolic categories: (a) poor metabolizers which lack functional enzymes due to deficient allele inheritance; (b) intermediate metabolizers, that are heterozygous with one deficient allele or carrying two reduced activity alleles; (c) extensive metabolizers that have normally functional enzymes due to wild type alleles inheritance, and (d) ultra-rapid metabolizers, which carry more than two copies of active alleles [89,90,91]. Several studies have been focused on drug–drug interactions and genetic polymorphisms including CYP2D6, CYP2C9 and *CYP2C19* activities [92,93,94]. In particular, *CYP2C19* enzyme is involved in the hepatic metabolism of drugs such as chemotherapeutic agents (cyclophosphamide), anti-epileptics (S-mephenytoin, diazepam, phenobarbitone), antiplatelets (clopidogrel), proton pump inhibitors (omeprazole, pantoprazole, lansoprazole, rabeprazole), antivirals (nelfinavir), and antidepressants (amitriptyline, clomipramine) [95,96,97].

The major CYP isoenzymes have been characterized at the molecular level and their different substrates, inhibitors, and inducers have been identified [98]. Drug interactions can occur at a pharmacokinetic level—involving the absorption, distribution, and elimination of the drug [27]. There are four different terms were used in drug–drug interactions. (I) pharmacogenetics refers to how variation in one single gene influences the response to a single drug [99], (II) pharmacogenomics refers to how all of the genes (the genome) can influence responses [100], (III) pharmacokinetic interactions, in which one drug interferes with the disposition of another, alter the concentration of the drug at the site of action [101], and (IV) pharmacodynamic interactions result in a modification of the pharmacologic action of a drug without an alteration in its plasma or central nervous system (CNS) concentration [27]. Drug interactions are particularly common in the treatment of patients with epilepsy. As shown in Figure 1, pharmacokinetics describes how the body interacts with drugs, including absorption, distribution, metabolism and excretion. There are several factors can influence pharmacokinetic parameters such as hepatic and renal disease, decreased absorptive capacity, route of administration, impaired blood flow, variability in metabolizing enzyme activity, drugs, certain foods, gender and age [102].

To better understand the genetics and drug metabolizing enzymes of *CYP2C19* it is important to study the variability in drug response and adverse drug reactions [103]. The genetic polymorphisms of these enzymes determine the variable internal exposure to the drug and its metabolites encompassing small insertions or deletions, SNP and variable copy numbers (CVNs) including gene deletion or amplification [104,105]. Subjects with epilepsy can be classified based on the ability to metabolize *CYP2C19* substrates. For example, EM individuals are homozygous for the CYP2C19*1 allele, which is associated with functional *CYP2C19*-mediated metabolism. The IM genotype consists of one wild-type allele and one variant allele that encodes reduced or absent enzyme function (e.g., *1/*2, *1/*3), resulting in decreased *CYP2C19* activity [106], while, PM individuals have two loss-of-function alleles (e.g., *2/*2, *2/*3, *3/*3), resulting in markedly reduced or absent *CYP2C19* activity [106]. For *CYP2C19* (rs12248560), SNP is located on the CYP2C19*17 allele and is a C > T transition in the promoter that creates a consensus binding site for the GATA transcription factor family, resulting in increased *CYP2C19* expression and activity [107,108]. It has been indicated that the CYP2C19*17 allele frequencies are approximately 21% in Caucasians, 16% in African-Americans, and 3% in Asians [83,108]. Individuals who carry one or two *17 gain-of-function alleles (e.g., *1/*17, *17/*17) may be categorized as UMs. However, the phenotypic consequences of a loss-of-function allele and a *17 compound heterozygous genotype (e.g., *2/*17) is currently unclear but may be in between the EM and IM phenotypes, and possibly substrate-dependent [109,110]. There was conflict about whether CYP2C19*1/*17 is considered an UM or EM in literature. Two studies reported about this CYP2C19*1/*17 metabolism, while, another considered those with the CYP2C19*1/*17 heterozygotes as UM [111], while, other considered them as EMs [112] within the same group as the wild-type CYP2C19*1 homozygotes. For *CYP2C19* (rs4244285) is the defining polymorphism of the CYP2C19*2 allele and is a synonymous G > A transition in exon 5 that creates an aberrant splice site, and this change alters the mRNA reading frame, which results in a truncated, non-functional protein [113]. This CYP2C19*2 allele is the most common *CYP2C19* loss-of-function allele, with allele frequencies of approximately 12% in Caucasians, 15% in African-Americans, and 29–35% in Asians [83]. The *CYP2C19* “poor metabolism” phenotype was initially discovered by studies on impaired mephenytoin metabolism and the major molecular defect responsible for the trait is the CYP2C19*2 (rs4244285) loss-of-function allele [113]. For CYP2C19*2 SNPs can cause a 40-nucleotide deletion and a frame shift which leads to a premature stop codon, result in the production of a truncated protein without enzymatic activity [114]. The amino acid positions for each of these SNPs are listed in Table 3.

For *CYP2C19* (rs4986893) is the defining polymorphism of the CYP2C19*3 allele and is a G > A transition in exon 4 that results in a premature termination codon at amino acid 212 [115]. The CYP2C19*3 allele frequencies in most populations is below 1%; however, it is more prevalent among Asians (2–9%) [83]. As shown in Figure 6, we identified the two up-and-down streams SNPs for each as well as their location on the genome (Figure 6).

The metabolic enzymes are well known for their contribution to drug–drug interactions and genetic polymorphisms, and the ultimate goal of drug metabolism is to get rid of the drug by generating highly water soluble, polar, and excretable metabolites [69]. For example, the CYP450 enzymes can play a major role in metabolic drug oxidation and accounts for approximately 75% of the total drug metabolism [105,116]. Furthermore, CYP450 enzymes are classified based on gene similarity (cytochromes within families have more than 40% homology in their protein sequence) [117]. CYP1, CYP2 and CYP3 are the major families of the CYP system involved in drug metabolism. Genetic polymorphism of the CYP enzymes affects their expression and is an important determinant of individual susceptibility to drug toxicity [118,119]. Poor *CYP2C19* metabolizers are found in 5% of white people, but in up to 20% of Asians [120]. In contrast, variants of CYP2C9 are more prevalent among whites (∼35%) compared with African-American and Asian populations (<10%) [121].

Genomic sequencing has been used in research in broad applications including rare disease and cancer. The transition from targeted gene sequencing whole exome sequencing to whole genome sequencing has only been made possible due to rapid advancements in technologies and informatics that have dropped the cost per base of DNA sequencing and analysis [122]. Advances in genomic technologies that will facilitate genome-wide discovery of both common and rare variants have led to a rapid increase in our understanding of epilepsy genetics.

## 8. Functional Genomics and Precision Medicine

The human genome project will continue to revolutionize the practice of medicine. The whole-genome sequence variation in an individual has shown that novel and rare nonsynonymous single nucleotide polymorphisms (SNPs) are more likely to affect protein function and cause phenotypic effects [123]. A variety of strategies are now being employed to take advantage of massively parallel DNA sequencing to yield new discoveries in epilepsy [124,125,126]. These include whole-genome sequencing (WGS) and whole-exome sequencing (WES) [127,128,129,130,131]. WAS is a powerful tool in the study of complex diseases, however, GWAS are underpowered, in general, to detect effects of rare and novel SNPs [132]. For example, a large GWAS in epilepsy including 3445 patients with focal epilepsy, showed no significance of genetic variants [133,134].

Next-generation DNA sequencing allows a near comprehensive analysis of genetic variants, has to date been applied to only a small cohort of sporadic (idiopathic generalized) epilepsy patients in one study and was restricted to exome sequencing; this limited study did not produce positive results [135]. The initial GWAS results suggest that genetic variation makes only a modest contribution to epilepsy susceptibility and are consistent with results in other human diseases of the nervous system [136]. The first association of genetic polymorphisms with the metabolism of an anti-epileptic drug has been reported in the association of variation in drug-metabolizing enzymes CYP2C9 and *CYP2C19* with phenytoin dose [137], providing hope for individualized therapy for patients. The whole-genome sequencing (WGS) on six patients with severe early-onset epilepsy was conducted [138], and other study were also conducted on structural genomic variation in childhood epilepsies with complex phenotypes [139].

**Figure 7:**
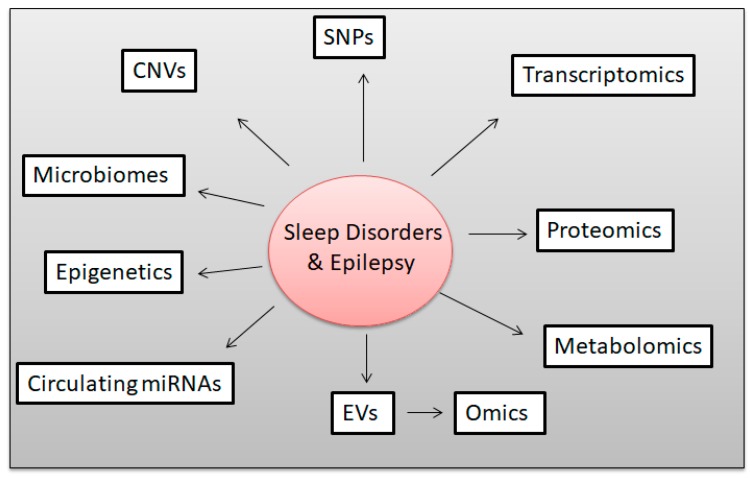
Diagram showing there is an urgent need for multiple disciplines to research children with sleep disorders and epilepsy to better understand the pathophysiology of the disease. This includes studying single nucleotide polymorphisms (SNPs), copy number variations (CNVs), transcriptomic mRNA, or the mRNA sequence, proteomics, circulating miRNA, microbiomes, and epigenetics. In addition, extracellular vesicles cargos which also contains DNAs, RNAs, miRNA and proteins.

Biomarkers such as DNA, RNA, proteins and metabolites can reflect an individual’s state of health or disease [140]. In addition to the SNPs and CNVs approaches as shown in Figure 7, we recommend a multiple research disciplines that involve epigenetics, transcriptomics, proteomics, circulating miRNAs and microtomic (OMICS). These technologies will lead to advances in understanding different genomic and genetic architectures across all the major classes of epilepsy, including uncovering a surprising overlap among seemingly different disorders. We will briefly discuss miRNAs because single miRNA can turn one or thousands of genes on or off which regulates gene expression and SNPs. MicroRNA is a class of small non-coding RNA that plays a key role in epigenetic regulation by controlling the translation and/or stability of mRNAs, mainly via sequence-specific binding within the 3′ untranslated region of mRNA transcripts. Several studies have reported the potential of miRNAs as biomarkers for different aspects of the epileptogenic process [141,142]. Recent studies have reported that the associations of miRNAs and SNPs, one of which in childhood of epilepsy [143], and the relation to drug-resistance [144]. Here, we show for the first time the number of miRNAs that targeting the *CYP2C19* gene, which includes the following: hsa-miR-1270, hsa-miR-455-3p, hsa-miR-4325, hsa-miR-7703, hsa-miR-6880-5p, hsa-miR-6851-5p, hsa-miR-3689d, hsa-miR-4683, hsa-miR-6499-3p, hsa-miR-767-5p, hsa-miR-6134, hsa-miR-4537, hsa-miR-5186, hsa-miR-665, hsa-miR-4459, hsa-miR-92b-3p, hsa-miR-92a-3p, hsa-miR-367-3p, hsa-miR-363-3p, hsa-miR-32-5p, hsa-miR-25-3p, hsa-miR-3180-5p, hsa-miR-3912-5p, hsa-miR-874-3p, hsa-miR-3157-3p, hsa-miR-5195-3p, hsa-miR-145-5p, and hsa-miR-6886-3p. Since, about 28 miRNAs can target the *CYP2C19* gene, we believe that during the disease status in children with epilepsy, or during treatment with phenobarbital, one or multiple miRNA may be involved, along with the regulation of the *CYP2C19* gene, which in turn up-regulates or down regulates the genes and also changes the genetic variations of the gene.

Several studies have highlighted that adipose tissue communicates systemically with other organs (brain, liver, skeletal muscle) and locally with other cells (preadipocytes, endothelial cells and monocytes/macrophages) through secreted products [145,146]. For example, cells can communicate with neighboring cells or with distant cells through the secretion of extracellular vesicles (EVs).

## 9. Extracellular Vesicles (EVs)

EVs are membrane-bound nanoparticles produced by most of all cell types and are part of mechanism of intercellular communication, a function of vital importance for multicellular organisms. These intercellular communications have been thought to be regulated by cell-to-cell contact and release of soluble molecules into the extracellular space, and these molecules covey the signal through their uptake or binding to specific receptors on target cells. Over the last decade, the interest in the role of EVs, particularly exosomes, in both physiological and pathological conditions has significantly increased. They are gaining recognition as multi-molecular messengers acting in both autocrine and paracrine ways modifying the activity and/or phenotype of recipient cells [147,148,149]. These EVs are composed of a lipid bilayer containing transmembrane proteins and enclosing cytosolic proteins and RNA, and the cells can secrete different types of EVs that have been classified according to their sub-cellular origin [147,148,149,150]. EVs are classified into three main types based on the size, shape and biogenesis. For example, exosomes are a class of EVs (50–150 nm in diameter) that originate from the endosome and are released from cells when multivesicular bodies (MVBs) containing intraluminal vesicles (ILV) fuse with the plasma membrane. While, microvesicles (also known as microparticles, shedding vesicles, or ectosomes; 200–1000 nm in diameter) are larger than exosomes and are released from cells through blebbing (budding out) and fission of the plasma membrane. Apoptotic bodies are large EVs (1000–5000 nm diameter) that are shed from cells during apoptosis [147]. Exosomes are proposed to have an important role also in pathogenic processes including autoimmune diseases, inflammation, metabolic and cardiovascular diseases [147,151,152,153]. Many studies demonstrated the role of exosomes in neuronal protection, regeneration and development, as well as synaptic plasticity. Accordingly, exosomes have been found released by neurons, astrocytes, oligodendrocytes, microglia, and neural stem cells [148,154,155]. Furthermore, exosomes have the capacity to cross the blood–brain barrier (BBB) making them excellent candidates for therapeutic interventions aimed at regenerating damaged CNS districts [148,155]. A major bottleneck in the advancement of exosome-based therapy into clinic is the development of high scale and efficient production of clinical-grade exosomes, and this would require sterile generation of exosomes with therapeutic payloads, produced in sufficient amounts for clinical testing, without batch-to-batch variation leading to compromised efficacy.

## 10. Sleep Disordered Breathing (SDB), Epilepsy and EVs

In pediatrics with SDB, we have shown in two different studies that plasma exosomes obtained before OSA treatment induce endothelial dysfunction in naïve endothelial cells. In obese or OSA children with evidence of endothelial dysfunction but not among those with a preserved endothelial function, their plasma exosomes induced marked in vitro and in vivo functional and structural alterations in naïve endothelium that are mediated by selective components of the exosomal miRNA cargo [156]. In the second study, we examined the potential contribution of circulating exosomes to blood–brain barrier (BBB) disruption in the context of pediatric OSA, we explored, using an in vitro BBB system, the effect of plasma-derived exosomes from children with polysomnographically determined OSA with evidence of neurocognitive deficits; age-, sex-, ethnicity-, body mass index *z*-score, and apnea–hypopnea index–matched children with OSA and no evidence of cognitive deficits; as well as control children without OSA or cognitive deficits [148]. Recent studies have reported a possible role of heat shock proteins (HSP) as neurotoxic in the brain [157]. Furthermore, Hsp60 has been increased in temporal lobe epilepsy in both animals and human subjects [158]. It has been shown that HSP as an abundant marker for exosomes [159]. In addition, EVs have unique features for the ability to cross biological barriers including the blood–brain barrier (BBB), and to modulate inflammation and immune responses [160,161,162,163]. We believe that the link between EVs and BBB let us suggest that EVs can be exploited into several different clinical applications ranging from biomarkers to therapeutic carriers including epilepsy.

Here, we highlighted several recent studies about SDB and epilepsy in children [7,13,164,165]. However, we didn’t find any article linking pediatrics SDB, epilepsy and EVs. Over the last a few years, several studies have described the relation between the EVs and epilepsy in relation to their miRNA cargo as a potential tool for diagnosis [166,167]. The first study described the changes in the cerebrospinal fluid miRNAs, including the exosomal miRNAs, in patients with temporal lobe epilepsy [166], and the second study reported an altered miRNA profile in plasma exosomes from temporal lobe epilepsy with hippocampal sclerosis [167]. A recent article about epilepsy and EVs in adults suggested that EVs show promise as biomarkers, treatments and drug targets for epilepsy [168]. It has been proposed that EVs may play a new therapeutics application in drug-resistant epilepsy [169].

## 11. Conclusions

Sleep disorders are associated with many diseases including epilepsy. The brain comprises a vast dynamic network of tens of billions of cells, which respond to fluctuating changes in the environment. Progress in the understanding of human genetic variability and technological progress has empowered robust assessment of hundreds of thousands of polymorphisms, covering the majority of common genetic variations. As a result, enormous breakthroughs in understanding the genetic architecture of many complex phenotypes have surfaced. Despite the expectations of pharmacogenetics research in epilepsy, few studies have provided conclusive results, and even fewer have been translated into clinical practice. Genomic variation of *CYP2C19* was considered in drug pharmacokinetic proteins, in drug target proteins and finally across multiple candidate genes, thus providing three broad hypotheses as sources, housing potential influential genomic variations. Metabolic drug interactions may have important clinical consequences. In the case of phenobarbital, these interactions are particularly common, due to the fact that many of these agents are potent inducers (or in some case, inhibitors) of the drug metabolizing enzymes and they are usually administered chronically, often in combination therapy. Ion channels are frequently mutated in epilepsy disorders and, are therefore, frequent targets of pharmacological manipulations to reduce or prevent pathologic hypersynchronous activity in the epileptic brain. Presently, gene panel testing is the favored choice for epilepsy genetic diagnosis due to the lower cost and higher coverage of the technology. However, as the price for and WGS and next generation sequencing (NGS) continues to decrease, they will soon be fully integrated into the diagnostic setting, providing a wider range of diagnostic options for clinicians to utilize. The use of NGS technologies is the future of genetic diagnosis. As further research is completed on epilepsy along with the identification of the causal variants associated with the disease, NGS will become the most viable diagnostic option. The future study of epilepsy should focus on integrating whole-genome sequencing variants and environmental factors into epigenetic change to understand the potential mechanism of epilepsy (Figure 7), which will allow clinics to apply the most effective therapy based on its individual genetics. We believe the application of precision medicine as a gold standard is the key for treatment and diagnostics for epilepsy in the near future. Pharmacologic treatment of the most common pediatric sleep disorders lacks evidence, and alternative methods, which have been proved to alleviate symptoms are preferred in most cases. It is now apparent that EVs participate in a range of physiological processes and represent an important intercellular communication mechanism. Additionally, the specificity of EVs and their functional cargos (proteins, different classes of non-coding RNA, DNA and lipids) will provide a new source of accessible biomarkers in both children and adults. Further studies are necessary to clarify the effectiveness of these precision medicine approaches in genetic epilepsies including EVs.

## Figures and Tables

**Table 1:** List of GABA receptors and their sequences homology, Kyoto Encyclopedia of Genes and Genomes (KEGG) pathways and gene ontology (GO).

Gene	Sequence Homology	KEGG Pathway	Gene Ontology
GABAR1 (NM_000806.5)	GAB type B receptor subunit 1 isoform X2, (XP_006715110.1), GAB type B receptor subunit 1 isoform X3 (XP_011512755.1), GAB type B receptor subunit 1 isoform a precursor (NP_001461.1), GAB type B receptor subunit 1 isoform X1 (XP_005249039.1), GAB type B receptor subunit 1 isoform b precursor (NP_068703.1), GAB type B receptor subunit 1 isoform k (NP_001305982.1), GAB type B receptor subunit 1 isoform X4 (XP_024302160.1), GAB type B receptor subunit 1 isoform X5 (XP_011512757.1), GAB type B receptor subunit 2 precursor (NP_005449.5), and GAB type B receptor subunit 2 isoform X1 (XP_016870820.1)	GABA A receptor activation, organism-specific biosystems, ion channel transport, organism-specific biosystem, ligand-gated ion channel transport, organism-specific biosystem, morphine addiction, organism-specific biosystem, neuronal system, organism-specific biosystem, and neuroactive ligand-receptor interaction, organism-specific biosystem	Cellular components: plasma membrane (GO:0005886), integral to plasma membrane (GO:0005887), membrane (GO:0016020), integral to membrane (GO:0016021), cell junction (GO:0030054), chloride channel complex (GO:0034707), and postsynaptic membrane (GO:0045211). Biological process: transport (GO:0006810), ion transport (GO:0006811), gamma-aminobutyric acid signaling pathway (GO:0007214), synaptic transmission (GO:0007268), ion transmembrane transport (GO:0034220), synaptic transmission, GABAergic (GO:0051932), transmembrane transport (GO:0055085). Molecular function: GABA_A_ receptor activity (GO:0004890), extracellular ligand-gated ion channel activity (GO:0005230), chloride channel activity (GO:0005254), protein binding (GO:0005515), drug binding (GO:0008144), and GABA receptor activity (GO:0016917).
GABBR1 (NM_000812)	GAB subunit beta-1 isoform X1 (XP_024309744.1), GAB receptor subunit beta-2 isoform 2 precursor (NP_000804.1), GAB receptor subunit beta-3 isoform 1 precursor (NP_000805.1), GAB receptor subunit beta-2 isoform 1 precursor (NP_068711.1), GAB receptor subunit beta-3 isoform 2 precursor (NP_068712.1), GAB receptor subunit beta-3 isoform 4 (NP_001178250.1), GAB receptor subunit beta-3 isoform X1(XP_011519730.1), GAB receptor subunit beta-3 isoform 3 (NP_001178249.1), GAB receptor subunit beta-1 isoform X2 (XP_016863474.1), and GAB subunit theta isoform X1 (XP_011529486.1)	GABAergic synapse, morphine addiction, neuroactive ligand-receptor interaction, nicotine addiction, retrograde endocannabinoid signaling, and serotonergic synapse	Cellular components: nucleus (GO:0005634), nuclear envelope (GO:0005635), cytoplasm (GO:0005737), plasma membrane (GO:0005886), and integral component of plasma membrane (GO:0005887). Biological process: ion transport (GO:0006811), chloride transport (GO:0006821), signal transduction (GO:0007165), gamma-aminobutyric acid signaling pathway (GO:0007214), and chemical synaptic transmission (GO:0007268). Molecular functions are GABA-A receptor activity (GO:0004890), ion channel activity (GO:0005216), extracellular ligand-gated ion channel activity (GO:0005230), anion channel activity (GO:0005253), and chloride channel activity (GO:0005254).
GABBR2, GABA-C (NM_002043.4)	GAB receptor subunit rho-2 isoform X1 (XP_011534015.1), GAB receptor subunit rho-2 isoform X2 (XP_011534016.1), GAB receptor subunit rho-1 isoform b precursor (NP_001243632.1), GAB receptor subunit rho-1 isoform a precursor (NP_002033.2), GAB receptor subunit rho-1 isoform c (NP_001243633.1), GAB receptor subunit rho-3 precursor (NP_001099050.1), GAB receptor subunit beta-3 isoform 1 precursor (NP_000805.1), GAB receptor subunit beta-3 isoform 2 precursor (NP_068712.1), GAB receptor subunit beta-1 isoform X1 (XP_024309744.1), and GAB receptor subunit beta-1 precursor (NP_000803.2).	GABAergic synapse, morphine addiction, neuroactive ligand-receptor interaction, nicotine addiction, and retrograde endocannabinoid signaling	Cellular components: plasma membrane (GO:0005886), integral component of plasma membrane (GO:0005887), membrane (GO:0016020), integral component of membrane (GO:0016021), and cell junction (GO:0030054). Biological process: ion transport (GO:0006811), chloride transport (GO:0006821), signal transduction (GO:0007165), gamma-aminobutyric acid signaling pathway (GO:0007214), and chemical synaptic transmission (GO:0007268). Molecular function: GABA-A receptor activity (GO:0004890), ion channel activity (GO:0005216), extracellular ligand-gated ion channel activity (GO:0005230), chloride channel activity (GO:0005254), protein domain specific binding (GO:0019904).

**Table 2:** List of exon and intron in *CYP2C19* sequences information.

exon	c.startExon	c.endExon	g.startExon	g.endExon	lengthExon	lengthIntron
1	1	168	5001	5168	168	12,184
2	169	331	17,353	17,515	163	169
3	332	481	17,685	17,834	150	4959
4	482	642	22,794	22,954	161	1161
5	643	819	24,116	24,292	177	38,498
6	820	961	62,791	62,932	142	22,199
7	962	1149	85,132	85,319	188	6892
8	1150	1291	92,212	92,353	142	2674
9	1292	0	95028	95,209	182	

**Table 3:** List of single nucleotide polymorphisms (SNPs) involved in clinical drug metabolism in children with epilepsy.

Chr. Position	mRNA Position	dbSNP rs#	Allele Name	Heterozygosity	MAF	Function	dbSNP Allele	Protein Residue	Amino Zcid Position
94762706	1	rs28399504	CYP2C19*4	0.004	0.0008	missense	G	Val [V]	1
94775165	276	rs17878459	CYP2C19*17	0.046	0.009	missense	C	Asp [D]	92
94780653	636	rs4986893	CYP2C19*3	0.011	0.0142	nonsense	A	Trp [W]	212
94781859	681	rs4244285	CYP2C19*2	0.302	0.2214	synonymous	A	Pro [P]	227
94852738	1297	rs56337013	CYP2C19*5	0		missense	T	Trp [W]	433
